# Application of Imaging Examination Based on Deep Learning in the Diagnosis of Viral Senile Pneumonia

**DOI:** 10.1155/2022/6964283

**Published:** 2022-05-31

**Authors:** Xiaohui Deng, Xiaozhu Ge, Qiang Xue, Hongzhi Liu

**Affiliations:** ^1^Department of Geriatrics, Beijing Jishuitan Hospital, Beijing 100035, China; ^2^Tuberculosis Department, Shanxi Linfen Third People's Hospital, Linfen City, Shanxi Province 041000, China

## Abstract

Medical image classification technology, preferably which is based on the deep learning, is not only a key auxiliary diagnosis and treatment method in clinical medicine but also an important direction of scientific research. With the intensification of social aging, the incidence of viral elderly pneumonia has been on the rise and needs dedication from the research community. Doctors rely on personal theories and experience to use traditional methods to check the computed tomography (CT) images of the lungs of elderly patients one by one, which is likely to cause diagnosis errors. The accuracy of the traditional method certainly meets the clinical needs, but it has higher requirements on the theory and experience of medical staff, and the classification efficiency is low. Constructing an accurate and fast auxiliary system can effectively save medical resources. In response to the above problems, we have proposed a viral pneumonia diagnosis method for lung CT images, which is based on the convolutional neural networks. The main research work is carried out around the following aspects: First, in the lung CT image classification task, the traditional methods are inefficient and effective for doctors. The basic quality requirements of the model are high, or, in the model training, the effective training data are small, and so forth, causing problems such as model overfitting. A lung CT classification model based on the improved Inception-ResNet is proposed. In this model, first the overall architecture of the network model is designed, and then the Contrast-Limited Adaptive Histogram Equalization (CLAHE) algorithm is used to perform the same image enhancement processing on the dataset and data needed in this article, and then the pictures pass through three different network models. A binary classification study was carried out on viral pneumonia and normal lung images, and finally the accuracy, sensitivity, and specificity of the three models were compared. The experimental results show that the accuracy of the three models for the judgment of viral pneumonia is more than 95%. Among these, the model proposed in this article has better classification effect and fit, the highest accuracy rate, and less parameters and can be used for rapid screening of viral senile pneumonia. *Objective*. To complete the classification of lung CT images of the elderly with viral pneumonia based on the improved Inception-ResNet network architecture. *Methods*. (1) Find and study domestic and foreign medical literature, understand the diagnosis and treatment methods of viral pneumonia, and study lung CT imaging; compare the pattern classifications of deep learning in lung imaging at home and abroad, and further study the application of convolutional neural networks in the medical field application. (2) Study various models and technologies of convolutional neural networks, summarize them separately, and have in-depth understanding of convolutional neural networks, including architecture, methods, and related system frameworks, experimental environments, and so forth. *Results*. This paper proposes an optimized Inception-ResNet network architecture for image classification. The control experimental model uses two network models, GoogLeNet and ResNet, and selects the viral pneumonia dataset for training and testing. The experimental results are as follows: the sensitivity and specificity are superior to those in the other two models, which can be used for actual medical screening and diagnosis. *Conclusion*. The improved Inception-ResNet network model method in this paper performs better in terms of accuracy, sensitivity, and specificity. Every metric is higher than those in the ResNet model and the GoogLeNet model, improving the classification effect. In addition, it can be seen from the experimental results that the model used in this paper has a very good classification effect in the classification of new coronary pneumonia CT image data.

## 1. Introduction

Viral pneumonia is an inflammatory response caused by different types of respiratory viruses attacking the lung parenchyma through various means, including small airways, and alveoli. Usually, the virus spreads and develops downwards after infection in the nasal cavity and throat. It can also be caused by viremia caused by a virus latent in the body or various reasons (e.g., blood transfusion, organ transplantation, etc.). Viral pneumonia is mainly caused by direct infection through air (droplets) or contact (pollutants or patients) or caused by the onward spread of upper respiratory tract viral infections. Respiratory viruses are very contagious. They can cause regional outbreaks and global pandemics. They are increasingly becoming an important factor endangering global human health, especially the elderly's. It is a very important task to distinguish between the normal population and the viral pneumonia population quickly and accurately [1–3]. According to WHO estimates for 2012, around 450 million cases of pneumonia occur worldwide every year, causing 3 million deaths and accounting for 5.5% of overall mortality worldwide. It is the fourth cause of death worldwide and is particularly a serious threat to children and the elderly.

Viral infections, being more common, have been more extensively studied. In contrast, research into viral community-acquired pneumonia (CAP), despite its growing epidemiological significance in developing countries and in the pediatric population, has been limited. Assuming the rate of diagnosis to still be lower than real incidence, around 200 million cases of viral pneumonia occur annually throughout the world, half of which are in the elderly. Viral pneumonia is of great interest due to its impact on the elderly, its role as a facilitator of viral infections, and its ease of transmission, a factor which has transformed it into a worldwide threat.

In an important period of rapid development of science and technology in our country, the rapid development of IT technology, cloud computing technology, and data storage technology ushered in a new era of big data. Many aspects of our social activities, work, and life are strongly affected by big data. At the same time, with the advent of the era of big data, medical data resources have also been greatly improved, and medical data has become more and more sufficient. Many hospitals have integrated professional medical image information data systems (e.g., picture archiving and communication system/radiology information system (PACS/RIS) system, image archiving, equipment, consultation, appointment, management, charging, hospital management, and medical interactive information system) during many years of practice. For a single hospital database, for a long time, a large number of case resources kept by the hospital have accumulated over time, which has become a burden on the hospital database. In fact, it is a valuable data resource that has not been used. Most hospitals have one or more complete medical databases of their own, but the functions of the database are very streamlined. In most cases, they are used for daily basic operations, such as adding, deleting, changing, and checking [4–8]. With the large-scale increase of independent databases, it is easily apparent that the amount of data is large but the effective information is insufficient. Therefore, data mining and data analysis for medical images will become a common issue in academia and medicine. Because lung computed tomography (CT) images are 2D imaging, when examining lung diseases, false positive problems and medical equipment resource imbalances are often prone to problems. Increasing the detection rate, reducing false positives, and providing doctors with more accurate auxiliary judgments have become the current focus of lung disease diagnosis and disease management. Therefore, it is the current general trend to diagnose and predict lung diseases through artificial intelligence and big data environments. Different from traditional manual detection, deep learning is a computer that automatically learns image features, saves the learning model, and makes judgments based on the model. The learning speed, learning efficiency, and objectivity are beyond the reach of humans. In the case where the system environment is excellent enough, the machine can learn features and the learned model can be copied, which greatly improves efficiency; when the number of learning samples is large enough and accurate enough, the machine learning's judgment accuracy rate is also extremely high. Many theoretical studies and practices have shown that deep learning can be well applied to medical image processing and has ideal results in the diagnosis of gastric cancer and skin cancer. If samples can be collected and used for deep learning detection, the diagnosis efficiency will be greatly improved. This article is based on deep learning to diagnose viral pneumonia CT images of the elderly [9–14].

In response to the aforementioned issues, we have proposed a viral pneumonia diagnosis method for lung CT images, which is based on the convolutional neural networks. The main research work is carried out around the following aspects: First, in the lung CT image classification task, the traditional methods are inefficient and not effective for doctors. The basic quality requirements of the model are high, or, in the model training, the effective training data are small, and so forth, causing problems such as model overfitting. A lung CT classification model based on the improved Inception-ResNet is proposed. In this model, first the overall architecture of the network model is designed, and then the Contrast-Limited Adaptive Histogram Equalization (CLAHE) algorithm is used to perform the same image enhancement processing on the dataset and data needed in this article, and then the pictures pass through three different network models. A binary classification study was carried out on viral pneumonia and normal lung images, and finally the accuracy, sensitivity, and specificity of the three models were compared. The experimental results show that the accuracy of the three models for the judgment of viral pneumonia is more than 95%. Among them, the model proposed in this article has better classification effect and fit, the highest accuracy rate, and less parameters and can be used for rapid screening of viral senile pneumonia.

With the rapid development of deep learning image classification and segmentation technology at home and abroad, many domestic and foreign researchers have done a lot of exploration and practice on the deep learning technology of lung images. The following focuses on the research status of lung image detection based on deep learning at home and abroad. Nowadays, more and more medical research databases in our country and even the world have begun to open to the public for research, and the use of deep learning technology in medical images is also gradually increasing; in particular, the convolutional neural network among them has made a huge contribution to medical image processing. In order to obtain deeper data in medical pictures and improve the accuracy of judgment, researchers often choose deep learning technology to conduct research. The network structure of deep learning and medical image processing are systematically sorted out and used in medical diagnosis. Li et al. [15] used deep learning residual network to identify electrocardiogram (ECG) information and used Deep Residual Convolutional Neural Networks (DRCNN) algorithm with adaptive threshold filtering to distinguish ECG information and achieved high accuracy. Han et al. [16] used multimodal image information to learn and test Alzheimer's disease, constructed a network model based on convolutional neural network, and integrated the modal features to calculate the accuracy rate of more than 95%. Huang et al. [17] used an improved deep residual network to classify lung CT images, using the transfer learning model to reduce the demand for the amount of learning data, for deep learning with small samples. Liu et al. [18] selected the convolutional neural network model as the deep learning model to detect lung nodules classification method. It is worth noting that the article uses a special preprocessing of lung CT images, and the experiment achieved a final accuracy rate of 92.3%. Narin et al. [19] obtained 50 X-ray images from the coronary pneumonia database, plus 50 normal lung X-ray images from the general database, and constructed a small set of only 100 X-ray images, using ResNet50 residual network. The model performed image classification, and the accuracy of the experimental results was as high as 98%. Shi et al. [20] systematically reviewed the relationship between coronavirus-type pneumonia and deep learning, analyzed research papers about coronavirus-type pneumonia, and pointed out the significance of deep learning for image collection, segmentation, and diagnosis of coronary pneumonia. Wang et al. [21] established a type of coronavirus pneumonia model based on the CT image data of 723 cases of positive pneumonia and 413 cases of negative pneumonia. The combined UNet+ and ResNet50 model was applied to the diagnosis of coronary pneumonia, and the accuracy rate reached 97.4%. CT examination is currently a relatively advanced medical imaging technology, which can help imaging doctors conduct detailed analysis. It is of great significance for diagnosing coronary pneumonia and various lung diseases. Therefore, this article finally uses CT images of the lungs as they are very feasible to detect objects.

The purpose of this paper is to complete the classification of lung CT images of viral pneumonia in the elderly based on the improved Inception-ResNet network architecture. The control experimental model uses two network models, GoogLeNet and ResNet, and selects viral pneumonia datasets for training and testing for actual medical screening and diagnosis.

## 2. Proposed Deep Learning Based Method

### 2.1. Classification Network Based on Improved Inception-ResNet

Artificially distinguishing pneumonia CT requires the imaging department or respiratory doctors to distinguish one by one. The respiratory department is overcrowded, and doctors in other departments also need to learn to share the pressure, and the high workload will make doctors misjudge the condition. It is the general trend to use deep learning to improve the work efficiency of medical staff and put forward constructive reference opinions.

**Figure 1:**
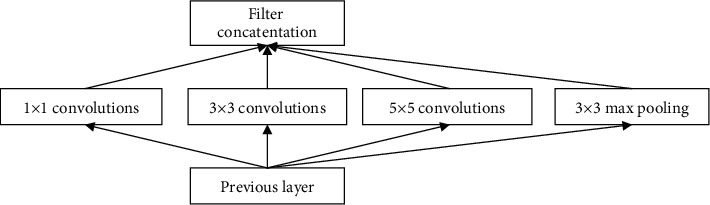
Structure diagram of the original version of the Inception module.

In convolutional neural network, the inception is widely used in the GoogLeNet. There are four generations of Inception modules, which are the original version, Inception-v1, Inception-v2/v3, and Inception-v4 [22–25]. The structure of the original version of the Inception module is shown in Figure 1.

**Figure 2:**
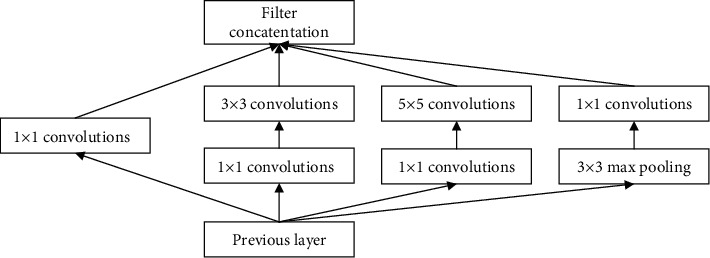
Inception-v1 structure diagram.

It can be seen that the Inception module outputs the feature map from a shallower network and then passes through three convolutional layers with different convolution kernel sizes and a pooling layer and finally superimposes and inputs it into a deeper network. However, the amount of calculation is very huge. Due to the need to reduce the amount of calculation, the Inception-v1 method was produced, as shown in Figure 2.

Inception-v1 has a 22-layer network structure. In order to make the output intermediate classification effect more obvious, every three Inception modules are equipped with an auxiliary classifier, thereby combining the models in disguise. Moreover, the information gradient of network response and propagation is strengthened, resulting in a better regularization effect.

**Figure 3:**
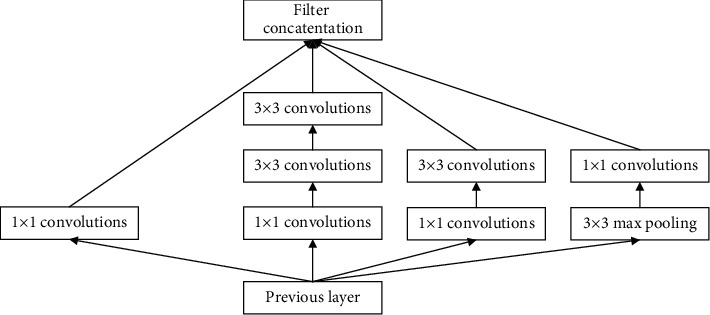
Inception-v2 structure diagram.

Inception-v2 is a model that improves the solution process of volume integration. Two consecutive 3 × 3 convolutional layers are used to replace a 5 × 5 convolutional layer, which enables a large number of experiments to prove that the data can be completely expressed, so that the data can be expressed more accurately in a deeper network. In addition, Inception-v2 also has a special structure, which is mainly used in high-dimensional features. Performing nonlinear mapping many times will result in more judgment signals, and the high-dimensional feature size is smaller and is easy to train, so it allows slightly broadening the network structure, as shown in Figure 3.

**Figure 4:**
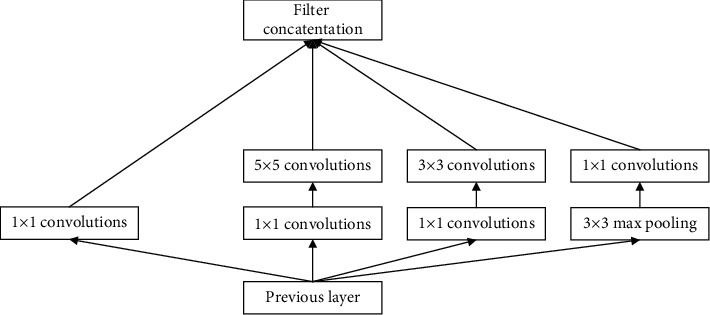
Inception-v3 structure diagram.

Inception-v3 uses RMSprop optimization algorithm and Label Smoothing Regularization (LSR) regularization model to improve the algorithm structure. In Inception-v3, GoogLeNet updated the v2 network structure again. Inceptionv3 used part of the network structure of v2, as shown in Figure 4.

The Inception method can use the volume base layer to reduce the number of channels of the model and adopt a different network for calculation to reduce the amount of calculation, so that the model can be used with a large amount of data. In order to consider the number of features and the amount of calculation at the same time, the Inception method uses the convolutional layer and the pooling layer to expand the calculation, and Inception has an auxiliary classifier, which can effectively alleviate the problem of gradient disappearance and ensure that the input and output image size is the same, while reducing the amount of calculation.

ResNet is a good model for classification, object detection, and image segmentation. The main features of ResNet are as follows: (1) a residual structure is proposed, and an ultradeep network structure with a breakthrough of 1000 layers is constructed; (2) a batch normalization method is used to accelerate training (dropout is discarded).

In order to integrate the advantages of GoogLeNet and ResNet to construct a fusion network, the pretrained Inception-ResNet has greater advantages. Inception-ResNet has the advantages of no pooling layer, fewer branches, rich feature information, and strong computing power.

**Figure 5:**
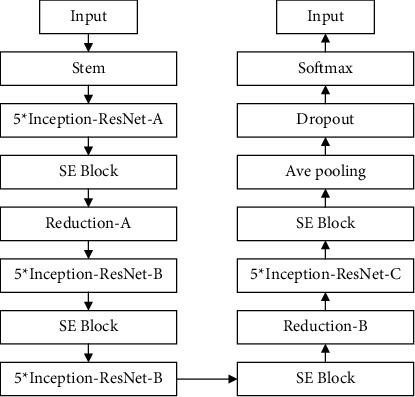
Schematic diagram of the model.

This paper combines the Inception and ResNet network structure and adds Stem, SE-Block, Reduction, and other parts. The overall network structure is as follows: (1) Change the size of the convolution kernel. Because the Inception structure is calculated through the convolution process, the original 7 × 7 and 5 × 5 convolution kernels were decomposed into 7 × 1, 1 × 7, 5 × 1, and 1 × 5 during the experiment. This can increase the running speed and double the depth of the network. (2) Introduce the residual structure. The purpose of introducing the residual structure of ResNet is to reduce the disappearance of gradients and overfitting problems caused by the increase in the number of layers. (3) Increase the feature weight and replace the activation function. The embedding of SE-Block is to increase the weight of the features in the network and to change the original activation function ReLU to LeakyReLU; the purpose is to increase the generalization ability of the network. (4) Confirm the number of categories. The last level is Softmax. There are 3 types of CT images of pneumonia in this article. Here, Softmax is set to 3. The detailed diagram of the model is shown in Figure 5.

**Figure 6:**
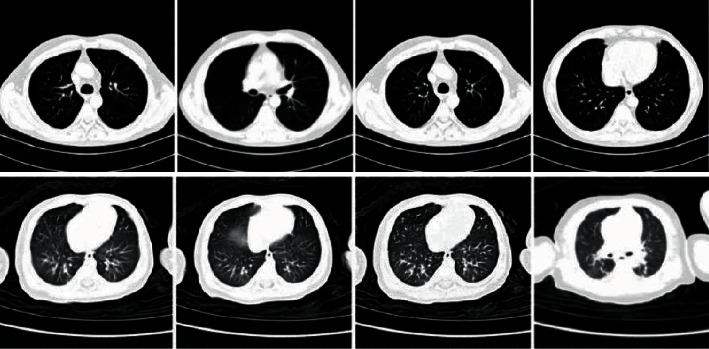
Normal lung images and viral pneumonia image.

The Inception-ResNet module designed in this paper is shown in Figure 6. The main steps are as follows: the first step is global average pooling, which will obtain global feature information from the image features of the inception network and output the result *x*; in the second step, the output from the previous pooling step *x* will pass through the fully connected (FC) layer, and the result *C*_1_*x* is obtained here; the purpose of this step is to reduce the number of channels of the output result of the first step, thereby reducing the calculation parameters; the third step is to pass the result of the previous step through the nonlinear layer (ReLU), set the activation function, and obtain the result *θ*(*C*_1_*x*); the fourth step is to pass the result to the fully connected (FC) layer and set the learned zoom parameter to *C*_2_; the purpose here is to restore the number of channels to the original number; the result obtained in this step is *C*_2_*θ*(*C*_1_*x*); the results of the four steps are transmitted to the nonlinear layer to obtain the weight information of each channel and output. All the feature information parameters are included in the fully connected layer, and the weight information is set by the activation function to achieve the purpose of focusing on learning important features and reducing interference items. The last step of the model is to superimpose the corresponding information from the ResNet module and the Inception module. In addition, in order to reduce the number of parameters and the amount of calculation, the residual module in this article is only applied to the last Inception module for combination, so that the amount of calculation is reduced without affecting the final accuracy of the experiment. The output function is as follows:

(1) $$\begin{array}{c} M=\sigma \left(C_{2}\theta \left(C_{1}x\right)\right). \end{array}$$

### 2.2. Loss Function

The loss function that is widely used in the calculation of classification is cross entropy. Standardize the two probability distributions *a* and *b*. Let *b* describe the cross entropy of *a* as

(2) $$\begin{array}{c} H\left(a,b\right)=-\sum a\left(x\right)\log\;b\left(x\right). \end{array}$$

The cross entropy satisfies the probability distribution function:

(3) $$\begin{array}{c} 0\leq xa\left(X-x\right)\leq 1,\sum a\left(X-x\right)=1. \end{array}$$

### 2.3. Data Preprocessing

The experimental data used in this article come from the dataset of the National Center for Biological Information (http://ncov-ai.big.ac.cn/download). Problems such as uneven size and lack of diversity of accurate and reliable lung CT datasets may hinder the use of convolutional neural networks for automatic diagnosis of new coronary pneumonia. The database we adopted perfectly circumvents this problem. This dataset has been certified by national professional research and is mainly a database of viral pneumonia cases and normal images. The distribution of the dataset is shown in Table 1. There are a total of 5000 CT images (2500 normal and 2500 viral), of which 1600 are used for training (total 3,200) and 400 are used for verification (total 800), each taking 500 sheets for testing (total 1000), and all images are in JPG format. Figure 6 shows the original images of three lung CTs. There are also big differences in the same type of CT images in the dataset. In view of the diversity of pictures of the same case, we use multiple acquisition methods and design a semiautomatic detection method through convolutional neural networks, and human input instructions are collected by the network model.

In order to reduce overfitting during the experiment and improve the accuracy of the network model, when collecting features in the early stage of the experiment, a data enhancement scheme is used to increase feature extraction, which mainly includes the following: adding random noise, mirroring, and rotation (90 degrees, 180 degrees, and 270 degrees).

## 3. Experiments and Discussion

### 3.1. Deployment Details

The experimental environment in this chapter is as follows: CPU is Intel i7-7700; memory is DDR4 16 G; GPU is NVIDIA RTX 2070, video memory is 8 G, operating system is Windows 10 system, and the experimental process mainly relies on GPU computing environment to run. In addition, this chapter uses the PyTorch deep learning framework to build the convolutional neural network of this article through the Python programming language. This article uses the Conda tool to configure the programming language software package and build the PyTorch software framework, reducing the problem of experimental failures caused by the Python version.

- The first step is to train the preprocessed training data for the network model.
- The second step is to use the loss function cross entropy to perform the network model during the training process.
- The third step is to transfer the test set to the network model. The results are tested and analyzed and adjusted accordingly. In this paper, the CLAHE algorithm is used to preprocess the dataset of chest CT images in the training set. Then the size of the CT image is set to 224 × 224, and then the CT image processed in the previous step is imported into the Inception-ResNet network model of this article to start training the model, and finally the data of the test set selected in this article are taken to the model for test experiments, and then the result information is recorded for comparison. The initial learning rate of this article is set to 0.001, and the initial weight decay value is set to 0.0001. When the learning rate decays more than 3 times, the weight decay is 0.

### 3.2. Evaluation Metrics

This article uses the three following indicators to objectively evaluate the model: accuracy (ACC), sensitivity (SEN), and specificity (SPE). The calculation formula is as follows:

(4) $$\begin{array}{c} \text{ACC}=\frac{TP+TN}{TP+TN+FP+FN},\\ \text{SEN}=\frac{TP}{TP+FN},\\ \text{SPE}=\frac{TN}{TN+FP}. \end{array}$$

### 3.3. Evaluation on Inception-ResNet

Table 2 shows the changes in the accuracy of the training set and the validation set using the Inception-ResNet network model. It is easy to find that the accuracy rate is basically stable after 5 iterations and maintains a relatively high level of about 95%. After 5 iterations, the accuracy rate has converged. Moreover, the accuracy of the first few iterations fluctuates significantly, mainly because the model is not sufficient for feature learning and cannot be well recognized.

### 3.4. Comparison with Other Models

First, the lung CT images are classified. The dataset used to classify the images includes a total of 5000 CT images. All images are subjected to the same preprocessing (compression, denoising, enhancement, etc.) and then sent to the convolutional neural network for model training. We set the test accuracy rate of the model to 98%. When the accuracy rate reaches or exceeds 98%, the training and verification process of this article is over, and then 1500 images (500 each) of the verification set where the model has not touched at all are used for the confusion matrix test. In order to evaluate the effectiveness of the model, this article performs a comparison with other convolutional neural networks, including ResNet18 and GoogLeNet, where all models use exactly the same image preprocessing algorithm, and then records and analyze the experimental prediction results. The experimental results of the three models are shown in Tables 3–5.

The improved Inception-ResNet network model method in this paper performs better in terms of accuracy, sensitivity, and specificity. Every index is higher than those in the ResNet model and the GoogLeNet model, and the classification effect is improved. In addition, it can be seen from the experimental results that the model used in this article has a very good classification effect in the classification of new coronary pneumonia CT image data.

## 4. Conclusion and Future Directions

In today's society, whether it is the existing computer technology, equipment upgrades, medical imaging technology, or even deep learning technology, all are flourishing and merging with each other, and the future can be expected. Based on the improved Inception-ResNet network architecture, this article has completed the classification of lung CT images of viral pneumonia in the elderly. The main contributions of this article are as follows: (1) Find and learn domestic and foreign medical literature, and understand the diagnosis and treatment methods of viral pneumonia; study lung CT imaging; compare the pattern classifications of deep learning in lung imaging at home and abroad, and further study the application of convolutional neural network in the medical field. (2) Study various models and technologies of convolutional neural networks, summarize them separately, and have in-depth understanding of convolutional neural networks, including architecture, methods, and related system frameworks, as well as experimental environments. (3) This paper proposes an optimized Inception-ResNet network architecture for image classification. The control experimental model uses two network models, GoogLeNet and ResNet, and selects the viral pneumonia dataset for training and testing. The experimental results are as follows: Sensitivity and specificity are superior to those in the other two models and can be used in actual medical screening and diagnosis. There are some limitations in this study. First, the sample size of this study is not large and it is a single-center study, so bias is inevitable. In future research, we will carry out multicenter, large-sample prospective studies, or more valuable conclusions shall be drawn.

This model can be further extended to analyze its effects on classification of other models' data specifically if the proposed model is combined with or integrated into other models.

## Figures and Tables

**Table 1:** Lung CT image dataset.

Class	Normal	Viral pneumonia
Training set	1600	1600
Validation set	400	400
Testing set	500	500
Total	2500	2500

**Table 2:** Inception-ResNet accuracy rate changes.

Epoch	Train_loss	Val_loss	ACC
1	0.115	0.105	0.951
2	0.108	0.099	0.954
3	0.105	0.094	0.954
4	0.095	0.086	0.957
5	0.090	0.084	0.958

**Table 3:** ResNet classification results.

Class	ACC	SEN	SPE
Normal	0.91	0.98	0.95
Viral pneumonia	0.97	0.90	0.99

**Table 4:** GoogLeNet classification results.

Class	ACC	SEN	SPE
Normal	0.89	0.96	0.94
Viral pneumonia	0.96	0.88	0.97

**Table 5:** Inception-ResNet classification results.

Class	ACC	SEN	SPE
Normal	0.92	0.99	0.96
Viral pneumonia	0.99	0.89	0.99

## Data Availability

The datasets used and analyzed during this study are available from the corresponding author upon reasonable request.
